# Impact of a methadone maintenance therapy pilot in Vietnam and its role in a scaled-up response

**DOI:** 10.1186/s12954-015-0075-9

**Published:** 2015-10-16

**Authors:** Tran Vu Hoang, Tran Thi Thanh Ha, Tran Minh Hoang, Nguyen To Nhu, Nguyen Cuong Quoc, Nguyen thi Minh Tam, Stephen Mills

**Affiliations:** Partners in Health Research, 47 Yen Phu Street, Tay Ho District, Hanoi, Vietnam; FHI 360, 7th floor, Hanoi Tourist Building, 8 Ly Thuong Kiet Street, Hanoi, Vietnam; Hanoi Medical University, No. 1, Ton That Tung Street, Hanoi, Vietnam; Vietnam Administration for AIDS Control, Vietnam Ministry of Health, 5th floor, 138a Giang Vo Street, Ba Dinh District, Hanoi, Vietnam; FHI 360, 19th Floor, Sindhorn Building, Wittayu Road, Bangkok, Thailand

**Keywords:** Methadone, Heroin, HIV, Antiretroviral treatment, Vietnam

## Abstract

**Background:**

As a dual response to the HIV epidemic and the high level of injecting drug use in Vietnam, the Ministry of Health (MOH) initiated a pilot methadone maintenance therapy (MMT) program in Hai Phong and Ho Chi Minh City (HCMC) in early 2009. The objectives of the pilot were to provide evidence on whether MMT could be successfully implemented in Vietnam and scaled up to other localities.

**Methods:**

A prospective study was conducted among 965 opiate drug users admitted to the pilot. Data on demographic characteristics, sexual behaviors, substance use behaviors (including heroin use), and blood-borne virus infection (HIV, hepatitis B, and hepatitis C) were collected at treatment initiation and then again at 3-, 6-, 9-, 12-, 18-, and 24-month intervals thereafter.

**Results:**

Twenty-four months after treatment initiation, heroin use as measured by urine test or self-report had reduced from 100 % of participants at both sites to 14.6 % in Hai Phong and 22.9 % in HCMC. When adjusted for multiple factors in Generalized Estimating Equations (GEE) logistic regression modeling, independent predictors of continued heroin use after 24 months of MMT in HCMC were the following: poor methadone adherence (adjusted odds ratio (AOR) = 3.7, 95 % confidence interval (CI) 1.8–7.8); currently on antiretroviral treatment (ART) (AOR = 1.8, 95 % CI 1.4–2.4); currently on TB treatment (AOR = 2.2, 95 % CI 1.4–3.4); currently experiencing family conflict (AOR = 1.6, 95 % CI 1.1–2.4); and currently employed (AOR = 0.8, 95 % CI 0.6–1.0).

For Hai Phong participants, predictors were the following: currently on ART (AOR = 2.0, 95 % CI = 1.4–3.0); currently experiencing family conflict (AOR = 2.0, 95 % CI = 1.0–3.9); and moderate adherence to methadone (AOR = 2.1, 95 % CI = 1.2–1.9). In Hai Phong, the percentage of participants who were employed had also increased by end of study from 35.0 to 52.8 %, while in HCMC the level remained relatively unchanged, between 52.2 and 55.1 %.

**Discussion:**

Study findings were used in multiple fora to convince policymakers and the public on the significant and vital role MMT can play in reducing heroin use and improving quality of life for individuals and families. Four years after this study was completed, Vietnam had expanded MMT to 162 clinics in 44 provinces serving 32,000 patients.

## Background

Methadone maintenance therapy (MMT) using oral methadone was first proposed as a favorable treatment for relieving heroin addiction over 50 years ago and has since been scaled up in numerous countries as an effective intervention for reducing heroin dependence and increasing quality of life [1]. With the advent of the HIV epidemic among people who inject drugs, evidence has shown that methadone increases adherence to antiretroviral therapy [2] and decreases mortality among people living with HIV [3]. MMT has also been shown to be associated with significant reductions in risk behaviors, e.g., injecting drugs, needle sharing, and having multiple sex partners or exchanging of sex for drugs or money [4]. In addition, MMT has been demonstrated to be a cost-effective treatment in several studies [5]. These findings as a whole point to MMT as an integral and essential component of both drug treatment strategies and HIV prevention and treatment strategies.

Since the early 1990s, the HIV epidemic in Vietnam has been predominantly fueled by the sharing of needles and other injecting equipment among people who inject drugs (PWID), mostly heroin [6–10]. HIV prevalence among this population remains as high as 40 % in many locations [11]. It is estimated by 2010 that there were more than 130,000 PWID nationwide in Vietnam [12], though this may be a significant underestimation [13]. Key HIV-related risk behaviors among this population include a continuing high frequency of injection, sharing of used needles and syringes, and unprotected sex with both regular and commercial sex partners [11, 14]. Efforts to improve drug and HIV prevention policies, the provision of harm reduction services, and outreach to rehabilitation services were then initiated in the early 2000s, [15]. However, one significant barrier to this was that the type of rehabilitation offered was predominantly a network of compulsory rehabilitation centers throughout the country that focused on a mixture of detoxification, labor, and lectures with little evidence-based service and a subsequent high rate of re-incarceration [16].

The next significant event was the MMT pilot discussed here. It was based in part on the observed reduction in heroin use and injection among drug users on MMT already documented in many countries in previous decades [17–20]. The objectives, again, were to provide evidence that MMT could be successfully implemented in Vietnam and scaled up to other localities.

In this paper, we examine the outcomes of this pilot, including prevalence of drug use, factors that predict continued drug use, methadone dosage patterns, and social factors such as employment and social relationships, among patients enrolled in a cohort study at six MMT outpatient clinics in Hai Phong and Ho Chi Minh City (HCMC), Vietnam. We also discuss both technical and policy implications using findings of this analysis.

## Methods

An observational prospective study was conducted among a cohort of participants who were consecutively enrolled in an MMT program in six clinics in Hai Phong and HCMC, Vietnam from January to October 2009. Under the Ministry of Health (MOH) regulations, people with the following characteristics were eligible: they must have been at least 18 years of age; they must have presented with opiate addiction for at least 3 years or more; and they must have voluntarily applied for MMT. Additional inclusion criteria were that participants were able to grant informed consent and had no other serious medical conditions that required hospitalization or extensive medical care.

A total of 965 patients enrolled in this study over the initial 9 months. After 2 years, 751 participants had completed the study and 214 had been lost to follow-up. Among those who were lost to follow-up, 16 had died, 97 had stopped MMT, 38 had withdrawn from the study, and 74 had been arrested. The absolute case number lost to follow-up at 12 months was 113 and at 24 months was 214, leaving 852 participants in the study at 12 months and 751 at the 24-month follow-up. The study retention proportion was 88.3 % (852/965) at 12 months and 77.8 % (751/965) at 24 months. Total study time was 20,058 person-months.

Each participant was followed up for 24 months, including study visits at enrollment and then at intervals of 3, 6, 9, 12, 18, and 24 months. At each visit, participants underwent an individual interview with a trained interviewer using a structured questionnaire. Information on treatment progress was also collected from patient medical records. Urine samples were collected to provide a biological marker of opiate use and participants were asked to provide 5-ml venous blood for detection of HIV, hepatitis B, and hepatitis C at each data collection interval.

The structured questionnaire for individual interviews included questions about participants’ characteristics (at baseline), drug use, sexual behaviors, living arrangements, employment, quality of life, and legal status including criminal activities. Questions on sexual and injecting risk behaviors were adopted from standardized questionnaires previously used for HIV/STI behavioral surveillance in Vietnam. Questions on injecting behaviors included history of injecting and detoxification, drug types and modes of use, frequency of use, and needle sharing. Sexual behavior questions covered sexual history, types and numbers of sex partners, and condom use. Timeframes of variables of interest depended on intervals between visits (3 months in the first year and 6 months in the second year). Questions were also asked on drug use in the 30 days prior to interview.

Participants’ quality of life was measured using a WHO module known as WHO QOL-BREF [21]. This tool has been widely used in health research in Vietnam and other countries [22, 23]. It includes 28 items measuring four domains of health: physical, psychological, social, and environment. Data were then extracted from patient clinical files, including assessment forms used in counseling sections with pre-structured data collection forms. Patients’ daily methadone doses were also taken from methadone dispensing databases.

The markers for heroin use were then derived from urinalysis. During treatment of patients, a series of urine tests was conducted by the clinic randomly at least once per month or by physician’s direction when drug use was suspected. Urine tests were also conducted at baseline, 3, 6, 9, 12, 18, and 24 months at study sites by trained technicians. The study team then used Instant-View Morphine (300) Urine Test (Dip-Strip) test kits (ALFA Scientific designs Inc.) for detection of morphine biomarkers.

To generate the variable of concurrent heroin use, a study subject was defined as continuing illicit heroin use if in the course of two intervals, they had at least one positive urine test (by random testing, by physician’s decision, or at initial interview) or if they self-reported continued drug use in the behavioral section of the interview questionnaire.

The study team used Abbott Determine ™ HBsAg test kits for detecting HBsAg and SD Bioline HCV test kits for identifying HCV antibodies, both in patient plasma. Specimens were collected by a technician at study site and transported to microbiology labs at preventive medicine centers in HCMC and Hai Phong.

HIV tests were performed following standard HIV diagnostic algorithms approved by the MOH. Screening was performed using Genscreen Ultra HIV Ag/Ab (Biorad, US) and then confirmed by rapid test (Determine HIV-1/2 (Alere, Japan)) and EIA test Murex HIV Ag/Ab (Dia Sorin, UK). The testing was conducted by lab technicians at provincial HIV/AIDS centers (PACs). The lab and technicians were certified by the National Reference Laboratory at the National Institute of Hygiene and Epidemiology (NIHE). Data were entered at study sites using Microsoft Access (Microsoft Inc, 2007), and data cleaning was performed after each round of study visits.

Data analyses were performed separately for respondents in Hai Phong and HCMC. Descriptive analyses were employed for participants’ social and behavioral characteristics. Trend analysis using random effects logistic regression was used to show changes in drug use by treatment period at intervals of 3, 6, 9, 12, 18, and 24 months after enrollment. Univariate analysis was then used to assess the association between heroin use (dependent variable) and other independent variables. Any independent variables that had a statistically significant association with the dependent variable (*p* value < 0.05) were included in the final model.

General estimating equation (GEE) logistic regression with random effects was used to take into account the fact that each participant was measured multiple times in the study for both dependent and independent variables. The GEE regression-based approach allowed for more comprehensive use of correlated data and for more reliable estimates in the study of substance use [24]. Variables significant from univariate analyses were added into the models using a stepwise procedure.

The study protocol, informed consent, and data collection forms were reviewed and approved by the FHI 360 Protection of Human Subjects Committee in North Carolina, USA, and by the Hanoi School of Public Health in Vietnam.

## Results

Demographic characteristics of study participants are presented in Table 1. Patients had used heroin an average of 10 years prior to initiating MMT (9.7 years among participants in Hai Phong and 9.6 years among participants in HCMC). More than 60 % of participants used heroin two to three times per day and one-third used it four times or more per day. At enrollment, 81.2 % of participants in Hai Phong and 87 % in HCMC were injecting. Just 15 % of participants in Hai Phong reported having ever being in mandatory detoxification centers, and 45.6 % of participants in HCMC reported having been in these centers at least once.

**Table 1:** Characteristics of MMT participants at baseline (*n* = 965)

	Hai Phong	HCMC	Total	*p* value
	% (*n*)	% (*n*)	% (*n*)	
Gender				
Male	98.1 (458)	92.0 (458)	94.9 (916)	0.000^b^
Female	1.9 (9)	8.0 (40)	5.1 (49)	
Age—mean (SE)	34.4 (0.1)	30.1 (0.1)	32.3 (0.1)	0.000^c^
Under 20	2.8 (13)	1.0 (5)	1.9(18)	0.000^d^
20 to 25	10.3 (48)	15.7 (78)	13.1 (126)	
25 to 29	19.1 (89)	45.9 (228)	32.9 (317)	
30 or more	67.9 (317)	37.4 (186)	52.2 (503)	
Education				
No school	0.6 (3)	0.8 (4)	0.7 (7)	0.631^d^
Primary school	8.8 (41)	10.6 (53)	9.7 (94)	
Secondary school	44.1 (206)	46.8 (233)	45.5 (439)	
High school	42.2 (197)	38.0 (189)	40.0 (386)	
College/university	4.3 (20)	3.8 (19)	4.0 (39)	
Monthly income—mean in US$ (SE)	285.5 (11.2)	292.4 (13.8)	289.1 (8.9)	0.699^c^
Duration of drug use—mean in year (SE)	9.7 (0.2)	9.6 (0.2)	9.7 (0.1)	0.596^c^
Frequency of heroin use at baseline				
Once per day	1.1 (5)	2.4 (12)	1.8 (17)	0.256^b^
2 to 3 times/day	62.9 (291)	63.6 (316)	63.2 (607)	
4 times or more per day	36.1 (167)	34.0 (169)	35.0 (336)	
Methods of administering drug in the 30 days prior to MMT enrollment				
Injecting	81.2 (379)	87.0 (433)	84.2 (812)	0.014^b^
Inhaling	21.6 (101)	18.7 (93)	20.1 (194)	0.253^b^
Had ever been in state-operated drug rehabilitation centers (“06 centers”)	15.0 (70)	45.6 (227)	30.8 (297)	0.000^b^
Had had sex with regular partners	46.7 (218)	43.4 (216)	45.0 (434)	0.302^b^
Had had commercial sex in the previous 3 months	3.6 (17)	2.6 (13)	3.1 (30)	0.357^b^
Had a regular sex partner who was also PWID	7.5 (35)	14.5 (72)	11.1 (107)	0.001^b^
HIV-positive	26.6 (124)	37.2 (146)^a^	31.4 (270)	0.001^b^
Hepatitis B positive	11.8 (55)	20.6 (102)	16.3 (157)	0.000^b^
Hepatitis C positive	40.0 (187)	69.8 (346)	55.4 (533)	0.000^b^

^a^In 392 participants (out of 498) who agreed to provide blood samples for HIV test or had HIV test results in their medical records at MMT clinics

^b^Chi-square test

^c^ *T* test

^d^Fisher’s exact test

At baseline, 26.6 % of participants in Hai Phong and 37.2 % in HCMC were HIV-positive (*p* < 0.01). The prevalence of HBV was 11.8 % in Hai Phong and 20.6 % in HCMC (*p* < 0.01). HCV prevalence was much higher among study participants: 40 % in Hai Phong and 69.8 % in HCMC (*p* < 0.01).

**Fig. 1:**
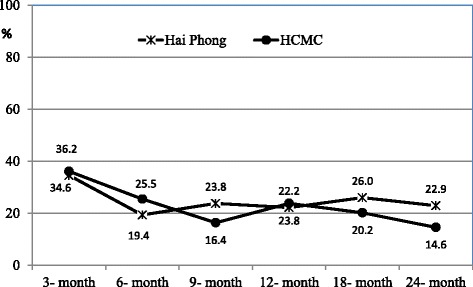
Trends in heroin use over time among participants

Figure 1 shows trends of concurrent heroin use among study participants. Where any patient had at least one of the following conditions in the 30 days prior to the study visit, “heroin use” was confirmed: (1) they self-reported drug use, (2) they tested positive for opiates in a urine test conducted by a clinician, or (3) they tested positive for opiates in a urine test conducted during a study visit.

The largest decreases in heroin use were observed in the period between the enrollment visit (baseline) and the 3-month visit. Among the entire body of participants using heroin at initiation of MMT, only 34.6 % in Hai Phong were still using it after 3 months, and in HCMC, 36.2 % of participants were still using after 3 months. This trend continued to the 6-month visit as well, with 19.4 % of participants in Hai Phong and 25.5 % in HCMC still using heroin in the 30 days prior to the 6-month follow-up interview. Then at the 24-month follow-up, 22.9 % of participants in Hai Phong and 14.6 % of participants in HCMC were still using heroin.

Table 2 shows the trends in methadone, antiretroviral treatment (ART), and tuberculosis (TB) treatment. After 3 months, the mean dosage of methadone among patients in Hai Phong was 9.6 ml, and in HCMC, this was 10.6 ml. There was a marked difference in dosing between Hai Phong and HCMC: the mean dosage among Hai Phong participants stabilized at 9.6 ml, where the mean among HCMC participants had increased from 10.5 ml at 3 months to 11.6 ml at 24 months. Adherence did decline in both cities: 18.4 % of participants reported missing one or two doses in the first 3 months, and by 24 months, this figure had increased to 41.4 % for the 6 months prior to final study visit. Percentage of patients who had missed three or four doses also increased from 1.0 % at 3 months to 3.7 % in the final 6 months. The data further showed that in the final 6 months, 3.7 % of participants had missed doses for a continuous 5 days or more.

**Table 2:** Methadone and ART

	Follow-up periods						*p* value
	0–3 months	4–6 months	7–9 months	10–12 months	13–18 months	19–24 months	
	(*n* = 930)	(*n* = 900)	(*n* = 871)	(*n* = 852)	(*n* = 802)	(*n* = 751)	
Mean methadone doses at study visits, in ml (SE)							
Hai Phong	9.6 (0.2)	9.9 (0.2)	9.5 (0.2)	9.5 (0.3)	9.4 (0.3)	9.6 (0.3)	0.248^a^
HCMC	10.6 (0.2)	11.0 (0.3)	11.2 (0.3)	11.4 (0.3)	11.5 (0.4)	11.6 (0.4)	0.000^a^
Total	10.1 (0.1)	10.4 (0.2)	10.4 (0.2)	10.4 (0.2)	10.4 (0.2)	10.6 (0.3)	0.000^a^
Missed methadone dose for 1–2 days, in %							
Hai Phong	19.5	23.6	30.7	41.1	48.4	47.7	0.000^a^
HCMC	17.3	22.0	26.5	28.1	33.8	34.7	0.000^a^
Total	18.4	22.8	28.6	34.6	41.3	41.4	0.000^a^
Missed methadone doses for continuous 3–4 days, in %							
Hai Phong	1.3	0.9	1.8	1.9	2.2	3.4	0.009^a^
HCMC	0.6	0.9	1.4	0.7	1.0	4.1	0.000^a^
Total	1.0	0.9	1.6	1.2	1.6	3.7	0.000^a^
Missed methadone doses for a continuous 5 days or more, in %							
Hai Phong	0.9	1.1	1.1	1.2	2.2	2.3	0.025^a^
HCMC	1.3	0.9	1.4	0.9	3.3	4.1	0.000^a^
Total	1.1	1.0	1.3	1.1	2.7	3.2	0.000^a^
ARV treatment, in %							
Hai Phong	14.2	12.9	15.1	14.0	16.3	16.0	0.202^a^
HCMC	39.8	50.8	35.9	38.7	41.2	43.3	0.846^a^
Total	27.2	31.9	25.5	26.3	28.4	29.2	0.827^a^

^a^Test for trend

At baseline visit, 27.2 % of participants were on ART, and by the end of month 24, this figure was 29.2 %. Notably, the proportion of patients who were on ART was higher in HCMC than it was in Hai Phong. At the end of the study follow-up period, 43.3 % of the HIV-positive patients in HCMC were receiving ARVs and 16 % of the HIV-positive patients in Hai Phong were receiving them.

Employment status and family characteristics are then presented in Table 3. In univariate analysis, factors that were statistically associated with concurrent heroin use were different for the two cities. In Hai Phong, they included having conflict in the family (odds ratio (OR): 2.0; 95 % confidence interval (CI): 1.0 4.0), higher methadone dosage (OR: 1.2; 95 % CI: 1.0–1.3), moderate level of adherence to methadone treatment (OR: 1.5; 95 % CI: 1.2–1.8), and currently on ART (OR: 2.2; 95 % CI: 1.5–3.1). In HCMC, more factors were found associated with concurrent heroin use among participants, including both protective and risk factors. Protective factors included age (OR: 0.8; 95 % CI: 0.7–0.9) and being employed full-time (OR: 0.7; 95 % CI: 0.6–0.9). Risk factors included having conflict in the family (OR: 1.9; 95 % CI: 1.3–2.7), living with injecting family members (OR: 1.7; 95 % CI: 1.0–3.0), higher methadone dosage (OR: 1.2; 95 % CI: 1.1–1.3), level of adherence in follow-up periods (for moderate adherence: OR: 1.5; 95 % CI: 1.2–1.9, for poor adherence: OR: 3.5; 95 % CI:1.7–7.1), currently on ART (OR: 2.1; 95 % CI 1.6–2.9), and currently on TB treatment (OR: 3.0; 95 % CI: 2.0–4.6) (Table 4).

**Table 3:** Participants’ social characteristics and behaviors

Variables of interest	Follow-up periods						*p* value
	0–3 months	4–6 months	7–9 months	10–12 months	13–18 months	19–24 months	
	(*n* = 930)	(*n* = 900)	(*n* = 871)	(*n* = 852)	(*n* = 802)	(*n* = 751)	
Percent of participants who were employed full-time and had stable monthly income (*n*)							
Hai Phong	35.0	47.4	49.0	54.4	51.6	52.8	0.000^a^
HCMC	52.2	57.9	61.5	56.1	53.2	55.1	0.705^a^
Total	43.8	52.7	55.2	55.3	52.4	53.9	0.002^a^
Percent of participants who reported having conflict within their family							
Hai Phong	2.0	3.8	2.8	2.3	0.7	0.8	0.005^a^
HCMC	17.3	3.1	4.2	2.6	6.1	3.9	0.000^a^
Total	9.8	3.4	3.4	2.5	3.4	2.3	0.000^a^
Percent of participants who reported having troubled relationships with family and community members							
Hai Phong	2.4	3.1	1.8	2.3	2.2	1.8	0.394^a^
HCMC	9.7	9.8	5.3	5.9	6.1	4.4	0.001^a^
Total	6.1	6.4	3.6	4.1	4.1	3.1	0.001^a^

^a^Test for trend

**Table 4:** Bivariate analyses of risk factors for concurrent heroin use among MMT patients

	Hai Phong		HCMC	
	Unadjusted OR	95 % CI	Unadjusted OR	95 % CI
Age (in 5-year units)	0.9	0.8–1.0	0.8	0.7–0.9
Gender (male is reference)	0.7	0.2–2.0	0.7	0.4–1.2
Ever been in an 06 center (yes/no)	1.0	0.7–1.5	1.1	0.8–1.5
Being employed full-time (yes/no)	1.1	0.9–1.4	0.7	0.6–0.9
Had problematic relation with family and/or community members (yes/no)	0.7	0.3–1.6	1.4	0.9–2.0
Had negative activities in family (yes/no)	2.0	1.0–4.0	1.9	1.3–2.7
Involved in criminal activities (yes/no)	1.2	0.6–2.2	1.6	0.6–4.2
Had PWID sex partners (yes/no)	1.2	0.4–3.6	1.3	0.9–1.8
Had PWID cohabitants (yes/no)	0.9	0.5–1.9	1.7	1.0–3.0
Methadone dosage (in 5-ml units)	1.2	1.0–1.3	1.2	1.1–1.3
Adherence to MMT^a^				
Good adherence	1	–	–	–
Moderate adherence	1.5	1.2–1.8	1.5	1.2–1.9
Poor adherence	1.9	0.8–4.5	3.5	1.7–7.1
Current ART (yes/no)	2.2	1.5–3.1	2.1	1.6–2.9
Current TB treatment (yes/no)	0.9	0.1–5.9	3.0	2.0–4.6

^a^Adherence level was defined as the following: good: no dose missed; moderate: missed doses for 1 to 4 continuous days; poor: missed doses for 5 or more continuous days

In the final model for Hai Phong (Table 5), independent predictors of continued heroin use were the following: currently on ART (adjusted odds ratio (AOR) = 2.0, 95 % CI = 1.4–3.0); currently experiencing family conflict (AOR = 2.0, 95 % CI = 1.0–3.9); and moderate adherence to methadone (AOR = 1.5, 95 % CI = 1.2–1.9).

**Table 5:** Multivariate analysis of factors associated with concurrent heroin use among MMT patients in Hai Phong

	Adjusted OR^a^	95 % CI
Had conflict in the family (yes/no)	2.0	1.0–3.9
Adherence to MMT		
Good	1	–
Moderate	1.5	1.2–1.9
Poor	2.1	0.9–5.0
ARV treatment (yes/no)	2.0	1.4–3.0

^a^Adjusted for age, gender, and methadone dose

In the final model for HCMC (Table 6), independent predictors of continued heroin use were the following: currently on ART (adjusted odds ratio (AOR) = 1.8, 95 % CI = 1.4–2.4); currently experiencing family conflict (AOR = 1.6, 95 % CI = 1.1–2.4); and moderate adherence to methadone (AOR = 1.7, 95 % CI = 1.3–2.2).

**Table 6:** Multivariate analyses of factors associated with concurrent heroin use among MMT patients in HCMC

	Adjusted OR^a^	95 % CI; *p* value
Age (in 5 year units)	0.8	0.7–0.9; 0.00
Being employed full-time (yes/no)	0.8	0.6–1.0; 0.05
Had conflict in the family (yes/no)	1.6	1.1–2.4; 0.03
Adherence to MMT		
Good	1	-
Moderate	1.7	1.3–2.2; 0.00
Poor	3.7	1.8–7.8; 0.00
ART (yes/no)	1.8	1.4–2.4; 0.00
TB treatment (yes/no)	2.2	1.4–3.4; 0.00

^a^Adjusted for gender, ever been in a rehabilitation center, had a PWID cohabitant or regular sex partner, problematic relationship with family or community members, and methadone dosage

In HCMC, independent predictors of continued heroin use after 24 months of MMT were the following: poor adherence (AOR = 3.7, 95 % CI 1.8–7.8); currently on ART (AOR = 1.8, 95 % CI 1.4–2.4); currently on TB treatment (AOR = 2.2, 95 % CI 1.4–3.4); currently experiencing family conflict (AOR = 1.6, 95 % CI 1.1–2.4); and currently employed (AOR = 0.8, 95 % CI 0.6–1.0).

## Discussion

This study showed that MMT among people who injected opioid drugs in the two cities led to improved quality of life in terms of reduced drug use, higher levels of employment, and reduced conflicts among families and communities. The reductions in drug use found in this study were comparable to that found in other studies [19, 25–29]. For example, in a recent cross-sectional survey in nine provinces in China, 27 % of patients on MMT were found to be still using opiates after 2 years of MMT, compared to 20 % in this study [30].

The data also showed high prevalence of HIV among MMT patients, at 26.6 % in Hai Phong and 37.2 % in HCMC. Approximately half of the HIV-positive individuals in the Hai Phong sample and almost all of the HIV-positive individuals in the HCMC sample were on ART. In both cities, patients who were on ART were more likely to continue to use heroin compared to patients not on ART. This may be due to the fact that ART allows very ill patients to return to their previous lifestyles, which in this case includes drug use. ART can also decrease methadone levels, and so, this may have also led to increased recidivism to drug use among patients on ART whose methadone dosages were not adjusted [31].

Several studies have shown the relationship between methadone dosage and drug use behaviors, with higher dosages associated with lower drug use [25, 32, 33]. However, in this study, no relationship between concurrent heroin use and methadone dosage was actually found. The results strongly suggest the need for appropriate clinical guidelines for methadone dose determination, however, particularly for patients who are also on ART.

Similarly, the data showed that TB treatment also increased the likelihood of continuing heroin use among MMT patients in HCMC. This is in agreement with findings from other studies, which show that rifampicin reduces the half-life of methadone, that TB is common among drug users who are HIV-positive, and that there is a need to consider TB status and treatment in determining methadone dosage for patients [34, 35].

The findings that patients’ adherence to methadone reduced with time in MMT and that poor adherence increased the risk of relapse to drug use and the likelihood of program withdrawal are supported by other studies [36]. Furthermore, the associations between adherence and heroin use, not only for those with poor adherence but also for patients who missed just one or two doses, strongly suggests that monitoring patient adherence will help to monitor treatment failure. An early warning system using data from methadone dispensing may be useful to provide necessary and timely support to concurrent heroin users. Future studies to understand patterns and reasons for non-adherence among people who use heroin are thus needed.

In HCMC, the finding that patients who had full-time employment and stable incomes also had a 20 % lower likelihood of continuing heroin use may be interpreted in two different ways: patients with stable jobs were more likely to stop heroin, or heroin abstainers were more likely to find a job. However, both interpretations indicate that MMT is more successful when it is supplemented with other social support services to help heroin users re-integrate into their communities.

Rates of HBV and HCV were both high in the samples in this study, with almost one in six infected with HBV and over half infected with HCV. These rates reinforce the need for both scaled-up HBV vaccination programs and scaled-up treatment management of both HBV and HCV among people who inject drugs (PWID), to monitor the onset and progression of liver disease and liver cancer.

The study had several limitations. Most notably, the sample of individuals consisted of patients selected to participate in a pilot program prior to a scaled-up response. Qualitative evidence from health providers suggests that these individuals may have been more motivated, had higher incomes, and come from more stable families than those in the general drug user population.

Males were also predominant in this study (98.1 % of the sample in Hai Phong and 92 % in HCMC), and this reflects existing estimates that the drug use population in Vietnam is over 95 % male [13]. Nevertheless, female drug use remains a critical issue in the country because it is highly related to sex work and HIV infection among sex workers. Because of the low sample size of females in this study, we were unable to make any conclusions regarding their methadone use and related outcomes among them. This remains a critical issue to address in further research.

Furthermore, the loss to follow-up of a sizeable portion of the cohort (22 %) also biased outcomes in an indeterminate way. Of the 214 individuals who were lost to follow-up, 74 of them had been arrested, however, representing over one-third of those who did not continue with the study. Yet, it was not possible to determine whether they were arrested for drug use or other reasons. However, the large number of arrests does suggest that increased cooperation with law enforcement is needed to ensure that PWID seeking and starting MMT are encouraged to continue treatment.

## Conclusions

The positive results of this study, combined with reports from individual families on the positive impacts of MMT on family stabilization and employment and qualitative evidence showing reductions in crime in areas where the pilot was located were the key in convincing policymakers to consider MMT as feasible and valuable, both for the control of HIV as well as drug use reduction. Study results were presented to policymakers at the provincial and national levels in numerous fora. A national media campaign called *Methadone is the Smart Solution: Health for Patients*, *Hope for Families*, *and Safety for Communities* used results from the pilot, family accounts, and positive community reactions to counter false perceptions of methadone and advocate for broader national and provincial support. A national policy was finally signed in 2011 approving the scale-up of MMT in all provinces, removing barriers to implementation and simplifying methadone licensing. By June 2015, Vietnam had expanded MMT to 162 clinics in 44 provinces serving 32,000 patients, with plans for continued scale-up.

This rollout of MMT throughout the country following advocacy and policy-level interventions that proactively used study results demonstrates the positive impacts of a carefully planned pilot of a sensitive new intervention and data on its effectiveness, safety, and impacts.
